# Exosomal microRNAs in Periodontitis: From Molecular Footprints to Clinical Frontiers

**DOI:** 10.1021/acsptsci.6c00155

**Published:** 2026-05-13

**Authors:** Maaz Anwer Memon, Danial Qasim Butt, Noriko Mizusawa, Muhammad Shahrukh Khan Sadiq, Wan Nazatul Shima Shahidan

**Affiliations:** † School of Dental Sciences, 65271 Universiti Sains Malaysia, Health Campus, Kubang Kerian, Kelantan 16150, Malaysia; ‡ Department of Oral Pathology, Shifa College of Dentistry, Shifa Tameer-e-Millat University, Islamabad 44000, Pakistan; § Department of Oral Bioscience, Institute of Biomedical Sciences, 241899 Tokushima University Graduate School, 3-Kuramoto-cho, Tokushima 7708504, Japan; ∥ Department of Oral Pathology, Bahria Dental College, 473284 Bahria University Health Sciences Campus, Karachi 75500, Pakistan; ⊥ Department of Pharmacology, School of Medical Sciences, Universiti Sains Malaysia, Health Campus, Kubang Kerian, Kelantan 16150, Malaysia

**Keywords:** exosomal miRNA, exosomes, miRNA, periodontitis, clinical

## Abstract

Periodontitis is a widespread chronic inflammatory disease driven by host–microbial interactions and immune dysregulation, often resulting in alveolar bone loss and systemic complications. Exosomes, small extracellular vesicles enriched with microRNAs (miRNAs), have emerged as central modulators of these processes, mediated intercellular communication, and influenced inflammation, immunity, tissue homeostasis, and host–pathogen interactions in the periodontal microenvironment. Existing literature has identified their diagnostic relevance; however, a comprehensive framework connecting molecular mechanisms to clinical applications remains lacking. This narrative review proposes an integrative conceptual framework that illustrates the progression from exosomal miRNA biogenesis to their regulatory roles in periodontal disease, linking microbial triggers, immune modulation, and their emerging clinical relevance. We trace the molecular footprints of these exosomal miRNAs, particularly miR-146a, miR-155, and miR-223, and assess their dynamic roles as both biomarkers and regulatory agents in inflammation and tissue remodeling. Recent analytical advances, including systems biology, multiomics integration, and network modeling, are examined for their potential to decode complex miRNA-mediated pathways. Integration of transcriptomic, proteomic, and exosomal data is highlighted as a promising approach to enhance mechanistic insights and predictive accuracy. We also evaluate emerging diagnostic and therapeutic applications of exosomal miRNAs, particularly their potential in noninvasive biomarker development and targeted regenerative strategies. Key translational challenges are addressed, including the lack of standardized isolation protocols, interindividual biological variability, and the need for *in vivo* validation. Future directions should prioritize the development of engineered exosomes for targeted miRNA and anti-inflammatory delivery, alongside exosomal miRNA-based biosensors for real-time, minimally invasive disease monitoring. By bridging molecular footprints with emerging analytical approaches, this review offers a forward-looking perspective on the translational potential of exosomal miRNAs in periodontology, demonstrating their potential in advancing precision diagnostics and targeted therapeutics.

## Graphical abstract

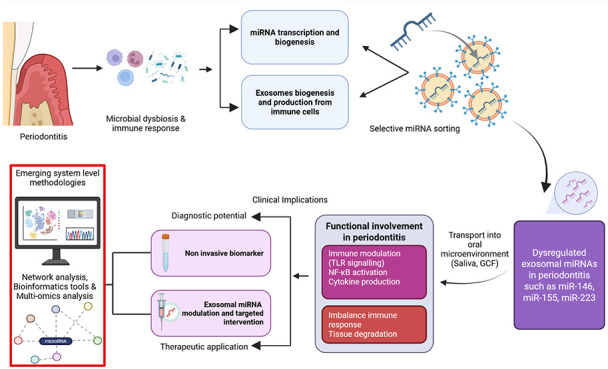

Periodontitis, a chronic inflammatory disease affecting approximately 20% to 50% of adults globally, leads to tissue destruction, including alveolar bone loss and periodontal ligament degradation.^1^ It is a widespread osteolytic inflammatory condition and has been demonstrated to exacerbate diseases such as cardiovascular disease (CVD), diabetes mellitus (DM), and rheumatoid arthritis (RA).^2–4^ It is characterized by inflammation within the periodontal tissues, resulting in the destruction of tooth-supporting structures, including the alveolar bone, and is a leading cause of tooth loss in adults.^5^ While the onset of periodontitis is initiated by the accumulation of a biofilm containing periodontal bacteria and bacterial virulence factors, its progression is contingent upon the activation of the host’s immune response.^6^ A central focus of contemporary periodontal research is the identification of effective diagnostic biomarkers that can influence clinical decision-making, improve patient outcomes, and advance healthcare strategies. The utilization of exosomal microRNAs (miRNAs) is significant for understanding how these vesicles modulate inflammatory cascades, and tissue regeneration at the molecular level is essential for advancing precision medicine approaches in periodontology.

Exosomes, membrane-bound extracellular vesicles ranging from 30 to 150 nm in diameter, have significantly advanced the understanding of intercellular communication by mediating the exchange of bioactive molecules, including proteins, lipids, and nucleic acids.^7^ Pan et al. (1985) were the first to characterize exosomes in differentiated reticulocytes, revealing that transferrin and other membrane-associated proteins are released in vesicular form as part of the reticulocyte maturation process.^8^ This discovery shifted the perception of exosomes from passive cellular debris to active participants in intercellular signaling, prompting extensive research into their biological functions.^9,10^

Among their cargo, miRNAs, a class of small noncoding RNAs ranging from 18 to 25 nucleotides in length, were first identified in the early 1990s following the discovery of *lin-4* in *Caenorhabditis elegans*.^11–13^ This gene regulates the timing of larval development by binding to complementary sequences in the 3′ untranslated region (UTR) of its target mRNA.^14^ The discovery of miRNAs as central regulators of gene expression initiated a paradigm shift in molecular biology.^15^ These small noncoding RNAs regulate gene expression post-transcriptionally by binding to target mRNAs, resulting in mRNA degradation or the inhibition of translation.^16^ Since then, miRNAs have been implicated in numerous biological processes, including apoptosis, differentiation, and immune responses.^17^

In response to periodontal inflammation, exosomes are released by diverse cell types, including gingival fibroblasts, immune cells, and periodontal ligament cells, contributing to intercellular signaling.^18^ These vesicles encapsulate a dynamic cargo of proteins, lipids, and miRNAs, shaping the cellular responses of recipient cells.^19^ Owing to their stability in body fluids, exosomes and their miRNA cargo represent promising noninvasive biomarkers for early disease detection and monitoring.^20^ Furthermore, their functional roles in immune regulation and tissue repair open new avenues for exosome-based therapeutics to modulate the disease progression. Compared to traditional biomarkers such as inflammatory cytokines or bacterial DNA,^21,22^ exosomal miRNAs offer enhanced specificity and mechanistic insight.^23,24^ Despite their potential, significant challenges persist, such as the necessity for standardized isolation protocols, *in vivo* functional validation of exosomal miRNAs, and the optimization of delivery strategies for therapeutic applications. Overcoming these limitations is essential for advancing exosome-based technologies from the research to clinical implementation.

Exosomal miRNAs play pivotal roles in modulating immune and inflammatory responses across a range of chronic conditions by regulating cytokine production, immune-cell differentiation, and tissue homeostasis. A recent study highlights the role of exosomal miRNAs in RA, emphasizing their regulatory impact on inflammation and immune cell function, and providing a comparative framework for understanding exosomal miRNA roles in chronic inflammatory diseases.^25^ While existing reviews on exosomal microRNAs in periodontitis have contributed valuable insights, particularly their diagnostic relevance, many remain descriptive and lack an integrative framework that connects molecular mechanisms with translational potential.^26,27^ Therapeutic implications and advanced methodologies for miRNA network analysis are still emerging areas that require deeper exploration. This review aims to advance the field by presenting a cohesive conceptual model that traces the molecular roles of exosomal miRNAs and examines their progression toward clinical application, integrating recent evidence on exosomal miRNAs in periodontitis and innovative analytical perspectives.

## 1 Periodontitis: A Molecular and Immune-Mediated Disease

Periodontitis is a chronic, immune-mediated inflammatory disease marked by progressive destruction of the periodontal ligament (PDL), cementum, and alveolar bone.^28^ It is initiated by the accumulation of a dysbiotic subgingival biofilm; among its microbial constituents, specific periodontal pathogens, particularly the “red complex” bacteria, including *Porphyromonas gingivalis* (*P. gingivalis*), *Prevotella intermedia*, and *Treponema denticola*, as described by Socransky, play an established role in disease initiation and progression through virulence factors that disrupt host–microbe homeostasis.^29^ Thus, both microbial dysbiosis and the host’s immune dysregulation act in synergy to drive disease onset and progression. However, the severity and progression of the disease are largely dictated by the host’s immune response rather than the microbial load alone.^30^ The immune system attempts to control the microbial challenge but simultaneously contributes to tissue damage through sustained inflammation.^31^

The innate immune response initiates through pattern recognition receptors (PRRs) such as Toll-like receptors (TLRs) and nucleotide-binding oligomerization domain-like receptors (NOD-like receptors), which activate nuclear factor kappa-light-chain-enhancer of activated B cells (NF-κB) and mitogen-activated protein kinase (MAPK) signaling pathways.^32,33^ This leads to the release of pro-inflammatory cytokines, including interleukin-1 beta (IL-1β), interleukin-6 (IL-6), and tumor necrosis factor-alpha (TNF-α), along with chemokines like interleukin-8 (IL-8) that recruit immune cells.^33,34^ Neutrophils and macrophages are rapidly mobilized; though essential for microbial clearance, their hyperactivation results in collateral damage via reactive oxygen species (ROS), proteolytic enzymes, and inflammatory mediators.^35^

As the disease advances, the adaptive immune response becomes more important. CD4+ T helper (Th) cells, including Th1, Th2, Th17, and regulatory T cells (Tregs), exerted distinct effects. Th17 cells promote osteoclastogenesis and bone resorption by releasing interleukin-17 (IL-17) and upregulating receptor activator of nuclear factor kappa B ligand (RANKL), while Tregs help mitigate inflammation.^36^ B cells and plasma cells also infiltrate the lesion, producing antibodies and cytokines, such as IL-6 and RANKL, which further contribute to tissue degradation. The imbalance between pro-inflammatory and anti-inflammatory signals results in a nonresolving inflammatory state, favoring tissue breakdown over repair.^4,37^ Beyond microbial and immune factors, genetic susceptibility significantly contributes to periodontal disease risk.^38,39^ Studies have identified single-nucleotide polymorphisms (SNPs) in genes encoding pro-inflammatory cytokines and host defense molecules, including TLR and the vitamin D receptor (VDR), which are associated with disease occurrence and severity. Importantly, the prevalence and functional impact of these polymorphisms differ across populations, highlighting population-specific genetic risk factors that modulate individual susceptibility to periodontitis.^40^ Recently, a study demonstrated that genetic risk extends beyond classical inflammatory pathways, with aggressive forms showing stronger associations than chronic periodontitis in Japanese populations and exhibiting distinct susceptibility profiles compared to Western cohorts.^41^ This genetic predisposition further supports the multifactorial nature of the disease, where host genetics interacts with microbial and environmental influences to shape clinical outcomes.

The persistent inflammatory burden imposed by periodontitis also extends beyond the periodontium, influencing systemic health.^42^ Circulating inflammatory mediators and microbial endotoxins may contribute to insulin resistance, endothelial dysfunction, and systemic inflammation, linking periodontitis to conditions such as DM, CVD, and RA.^42,43^ Within this immunopathogenic landscape, exosomal microRNAs have emerged as key modulators of immune and inflammatory pathways, offering a novel lens through which to explore molecular mechanisms and potential clinical applications.

## 2 Biological Basis of Exosomal miRNAs

### 2.1 Biogenesis of Exosomes

Exosomes are nanosized extracellular vesicles (40–160 nm) enclosed by a lipid bilayer, enriched in proteins, lipids, and nucleic acids that reflect their cell of origin.^44^ Their formation originates from the endosomal pathway in eukaryotic cells.^10^ Biogenesis begins with the inward budding of the plasma membrane to form early endosomes, which mature into multivesicular bodies (MVBs). Within MVBs, intraluminal vesicles (ILVs) develop through endosomal membrane invagination and serve as exosome precursors,^45^ as illustrated in Figure 1.

**Figure 1:**
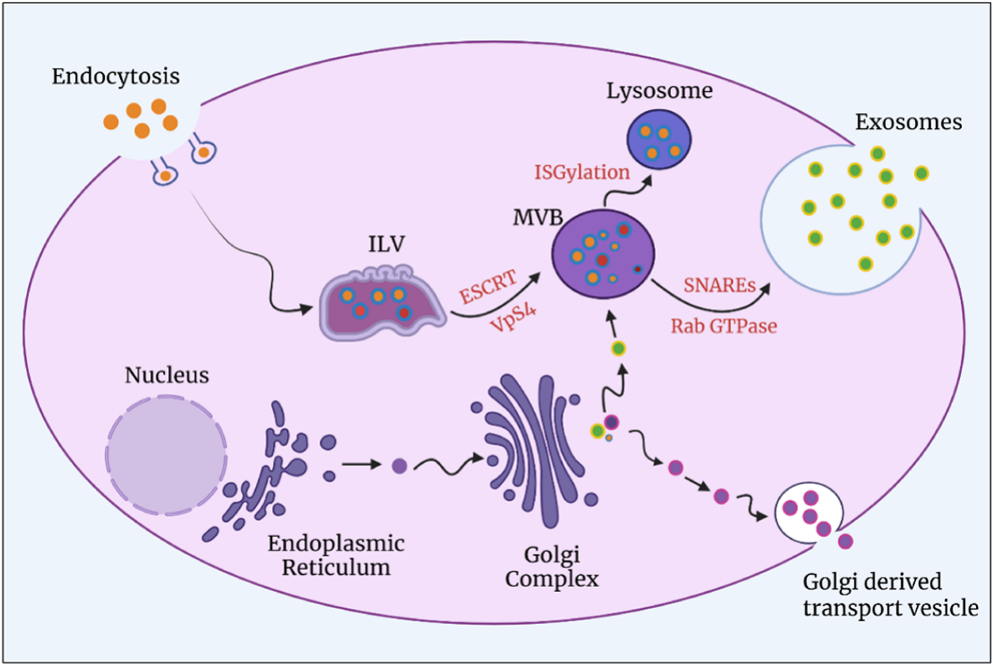
Exosomes originate from the endosomal system, where inward budding of the endosomal membrane forms intraluminal vesicles (ILVs) within early endosomes. These ILVs mature into multivesicular bodies (MVBs) through the action of endosomal sorting complex required for transport (ESCRT) complexes and Vacuolar Protein Sorting-associated protein 4 (Vps4). MVBs either fuse with lysosomes for degradation following ISGylation or with the plasma membrane to release exosomes, a process mediated by Rab GTPases and SNARE proteins. Golgi-derived vesicular transport also contributes to the pool of exosomal cargo. Created with BioRender.com.

This process is tightly regulated by the endosomal sorting complex required for transport (ESCRT), a system of four protein complexes (ESCRT-0, -I, -II, and -III) responsible for cargo selection and membrane remodeling.^10^ ESCRT-independent mechanisms also contribute, involving ceramides and tetraspanin-enriched microdomains.^46^ The transport and secretion of MVBs involve key regulators such as Rab GTPases (Rab27a/b), SNARE proteins (VAMP7), and cytoskeletal components like kinesins, dyneins, and actin-associated proteins, including cortactin.^45^ These coordinate the docking and fusion of MVBs with the plasma membrane, facilitating exosome release into the extracellular space.^47^

### 2.2 Exosome Formation and Cargo Loading Mechanisms

Cargo loading into exosomes is a selective and regulated process. The ESCRT machinery recognizes and sorts specific proteins, lipids, and nucleic acids into ILVs.^48^ RNA-binding proteins (RBPs), including heterogeneous nuclear ribonucleoprotein A2/B1 (hnRNPA2B1), Y-box-binding protein 1 (YBX1), and Argonaute 2 (Ago2), guide the selective incorporation of miRNAs based on specific sequence motifs.^49^ Post-transcriptional modifications, such as 3′ end methylation, and the activity of enzymes like neutral sphingomyelinase 2 (nSMase2), further modulate the sorting process, contributing to the preferential enrichment of certain miRNAs, such as miR-210 and miR-10b.^50–52^ This selective sorting ensures that exosomes carry cell-type-specific and stimulus-responsive molecular cargo, enabling them to mirror the physiological or pathological condition of their cells of origin.

### 2.3 Functional Roles of Exosomal miRNAs

Exosomes play a significant role in cell-to-cell communication by transporting biologically active cargo to the recipient cells. Once released, they can fuse with or be internalized by target cells, delivering their cargo and influencing cellular function.^10^ Among their diverse cargo, miRNAs are particularly important for regulating gene expression in recipient cells.^53^ These miRNAs modulate key biological processes, including immune responses, inflammation, tissue repair, and homeostasis.^54^ In periodontitis, exosomal miRNAs have been implicated in the regulation of key molecular pathways involved in periodontal inflammation, immune modulation, and alveolar bone remodeling.^55^

Exosomes are also characterized by their stability in body fluids, such as saliva, blood, and urine, due to their lipid bilayer that protects against enzymatic degradation.^54^ This stability supports long-distance communication, positioning exosomes as ideal carriers of noninvasive biomarkers. Their detectability in biological fluids enhances their clinical potential, and current techniques such as nanoparticle tracking analysis (NTA) and next-generation RNA sequencing support both basic research and potential diagnostics.^56^

## 3 microRNAs: Post-Transcriptional Regulators and Their Role in Inflammation

### 3.1 Biogenesis and Functional Mechanisms of miRNAs

miRNAs are small, noncoding RNAs approximately 18–24 nucleotides in length that play a significant role in post-transcriptional regulation of gene expression and cellular homeostasis.^57^ They are transcribed as primary miRNAs (pri-miRNAs) by RNA polymerase II and processed in the nucleus by the Drosha-DGCR8 complex into precursor miRNAs (pre-miRNAs). These pre-miRNAs are then exported to the cytoplasm via Exportin-5 and further cleaved by Dicer to form mature miRNA duplexes.^16^ One strand of the duplex is incorporated into the RNA-induced silencing complex (RISC), guiding it to target mRNAs through complementary base-pairing, primarily in the 3′ untranslated region (UTR).^58^ Depending on the degree of complementarity, miRNAs either induce mRNA degradation or inhibit translation, thereby fine-tuning gene expression and cellular function.^16^ The biogenesis of miRNAs follows a tightly regulated, multistep process involving nuclear and cytoplasmic components, as illustrated in Figure 2.

**Figure 2:**
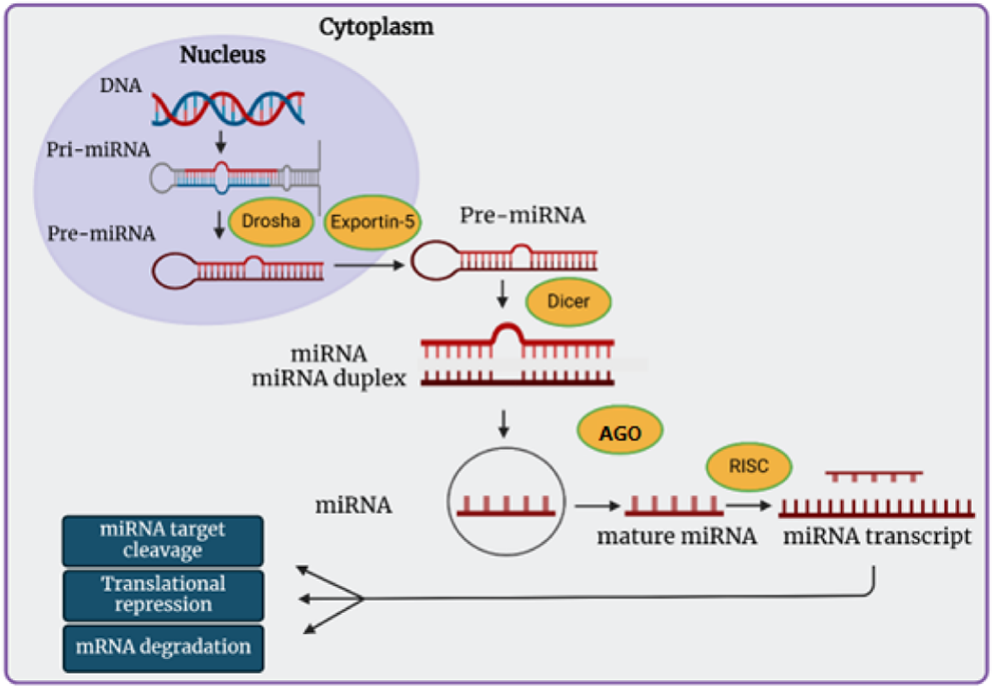
miRNA biogenesis pathway. miRNA is initially transcribed by RNA polymerase II to form primary miRNA (pri-miRNA), which is processed into pre-miRNA by the microprocessor complex, including Drosha. The precursor miRNA (pre-miRNA) is then exported to the cytoplasm via exportin-5, where it is cleaved by Dicer into a double-stranded miRNA. The double strand associates with the RNA-induced silencing complex (RISC), where the Argonaute protein separates the strands. The mature miRNA strand remains within RISC to regulate target mRNA, while the passenger strand is degraded. Created with BioRender.com.

### 3.2 Regulatory Role of miRNAs in Inflammation and Chronic Disease

miRNAs function as critical modulators of immune responses by regulating the expression of cytokines and chemokines, modulating receptor signaling pathways, and contributing to the resolution of inflammation.^59^ They play a central role in controlling the NF-κB signaling pathway, which is central to the initiation and maintenance of inflammatory responses.^60,61^ Additionally, miRNAs influence the differentiation and function of immune cells, thereby orchestrating both innate and adaptive immune responses.^62^ Exosomal miRNAs further mediate intercellular communication by delivering regulatory signals to distant cells, contributing to systemic immune modulation in inflammation and chronic diseases, including RA and inflammatory bowel disease (IBD).^63,64^

In RA, miR-146a and miR-155 are persistently upregulated in synovial tissue and exosomes, where they contribute to pro-inflammatory cytokine production.^65^ Conversely, miR-21 expression is markedly reduced in RA patients, a change associated with the disruption of the Th17/Treg balance, thereby exacerbating autoimmune responses and sustaining synovial inflammation.^66^ Additionally, miR-124a plays a critical role in RA pathogenesis by inhibiting the proliferation and invasion of rheumatoid arthritis synovial fibroblasts.^67^ Recent evidence highlights that these exosomal miRNAs form a core regulatory axis in RA pathogenesis, driving the production of pro-inflammatory cytokines, impairing the Treg/Th17 balance, and promoting the activation of synovial fibroblasts.^25^

In IBD, exosomal miR-223 contributes to the progression of IBD by modulating the intestinal epithelial barrier, resulting in the disruption of tight junction integrity.^68^ Exosomal miR-21, which is abundantly expressed in intestinal cells, represents one of the most significantly dysregulated miRNAs in active IBD, where it promotes pro-inflammatory cytokine production and compromises epithelial barrier function.^64^ Moreover, miR-31 is elevated in inflamed mucosa, where it attenuates inflammatory responses and reduces cytokine production while facilitating epithelial regeneration, thereby reflecting its dual role in both pathology and repair.^69^ Taken together, these exosomal miRNAs function as key regulators of immune responses across chronic inflammatory diseases, providing a comparative perspective on how their dysregulation drives immune imbalance and tissue destruction under conditions including periodontitis.

## 4 Exosomal miRNAs in the Pathogenesis of Periodontitis

### 4.1 Exosomal miRNA Profiles in Periodontitis

Recent studies have highlighted differential expression profiles of exosomal miRNAs in periodontitis, reflecting their involvement in disease progression and immune dysregulation. These miRNA signatures have been detected in exosomes isolated from saliva, plasma, and gingival crevicular fluid (GCF) of affected individuals.^55^ Among the exosomal miRNAs linked to periodontitis, several have been identified as key modulators of host immune responses and inflammatory signaling pathways. Table 1 presents key exosomal miRNAs in periodontitis, outlining their sources, roles, target pathways, and outcomes. Figure 3 provides a visual overview of exosomal miRNAs reported to be differentially expressed in periodontitis.

**Table 1:** Key Exosomal miRNAs Implicated in Periodontitis^a^

miRNA	Source	Role in pathogenesis	Target genes/pathways	Outcome	References
miR-146b-5p	LPS-stimulated hDPCs Exos	NF-κB signaling activated pro-inflammatory cytokine synthesis.	TRAF6, IRAK1, NF-κB signaling pathway	Inflammatory response ↑	^70^
miR-223	PDL-derived cells, inflamed gingival tissues Exos	Inhibits osteogenic differentiation in PDL-derived cells	TGFβR2, FGFR2	Osteogenic differentiation ↓	^71^
miR-155-5p	LPS-stimulated PDLSCs Exos	Modulates Th17/Treg balance and mitigates inflammation through the Th17/Treg/miR-155–5p/SIRT1 network.	SIRT1	Inflammatory response ↓	^72^
miR-21	hPDLSCs Exos	Suppressed by TNF-α, regulates adipogenic and osteogenic differentiation	Spry1	Adipogenesis ↑ Osteogenesis ↓	^73^
miR-381-3p	BMDM Exos	Inhibits NR5A2 and disrupts autophagy in GECs, exacerbating inflammation in diabetes.	NR5A2	Inflammatory cytokines ↑	^74^
miR-31-5p	NG-PDLSCs-Exo and HG-PDLSCs-Exos	Regulates alveolar bone regeneration and osteoclast differentiation	eNOS signaling pathway	Osteoclast differentiation ↓ Alveolar bone regeneration ↑	^75^
miR-126	HGFs Exos	Inhibits inflammation in periodontitis with diabetes by targeting TRAF6	TRAF6, IL-6, TNF-α, CCL2	Inflammation in diabetic periodontitis ↓	^76^
miR-200c	HGFs Exos	Attenuates inflammation and alveolar bone resorption in periodontitis	IL-6, IL-8, IFRD1, p65, p50, receptor activator of nuclear factor kappa-B ligand (RANKL), osteoprotegerin	Inflammation ↓	^77^
miR-221-3p and miR*-*222-3p	BMSCs Exos	Inhibition of osteogenic differentiation through IGF-1/ERK pathway	IGF-1, ERK Activation, Osteoblast Differentiation	Osteogenic gene expression Osteoblast differentiation ↓	^78^
miR-1260b	GMSCs Exos	Inhibits Wnt5a-mediated RANKL osteoclastogenic activity	Wnt5a, RANKL	Osteoclast differentiation ↓ Bone resorption ↓	^79^
miR-205-5p	PDLSCs Exos	Involved in alleviating inflammation and balancing Th17/Treg cells in chronic periodontitis	X-box binding protein 1 (XBP1)	Inflammation ↓	^80^

a Human dental pulp cells: hDPCs, periodontal ligament stem cells: PDLSCs, human periodontal ligament stem cells: hPDLSCs, normal-glucose-cultured PDLSCs: NG-PDLSCs-Exo, high-glucose-preconditioned PDLSCs: HG-PDLSCs-Exo, human gingival fibroblasts: HGFs, bone marrow mesenchymal stem cells: BMSCs, gingival mesenchymal stem cells: GMSCs Exo, bone marrow-derived macrophages: BMBD, TRAF6: TNF Receptor-Associated Factor 6, IRAK1: Interleukin-1 Receptor-Associated Kinase 1, TGFβR2: Transforming Growth Factor Beta Receptor 2, FGFR2: Fibroblast Growth Factor Receptor 2, Th17/Treg: T helper 17/Regulatory T cells, SIRT1: Sirtuin 1, Spry1: Sprouty Homologue 1, NR5A2: Nuclear Receptor Subfamily 5 Group A Member 2, eNOS: Endothelial Nitric Oxide Synthase, CCL2: C–C Motif Chemokine Ligand 2, IFRD1: Interferon Regulatory Factor Domain Containing 1, RANK: Receptor Activator of Nuclear Factor Kappa-B Ligand, IGF-1: Insulin-like Growth Factor 1, ERK: Extracellular Signal-Regulated Kinase, Wnt5a: Wingless-Type MMTV Integration Site Family Member 5A.

**Figure 3:**
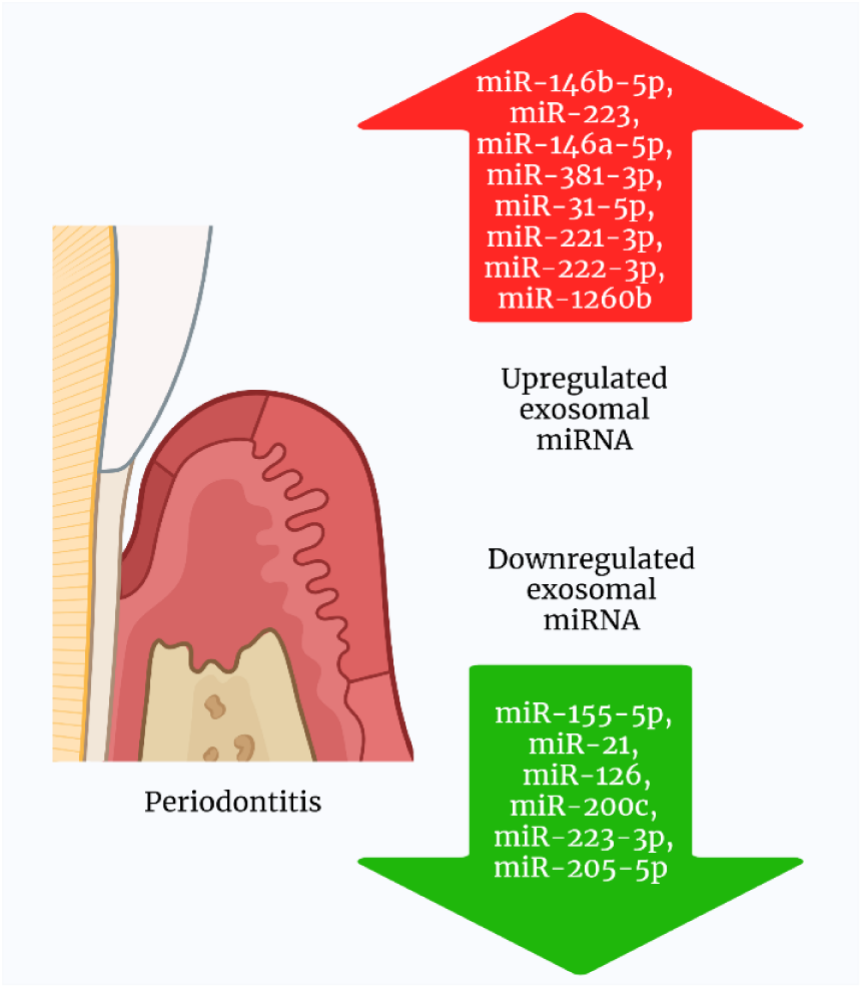
Exosomal miRNAs show differential expression between periodontitis patients and healthy controls, indicating their potential involvement in disease pathogenesis Created with BioRender.com.

### 4.2 Interaction with Immune Cells, Fibroblasts, and Osteoclasts

Exosomal miRNAs mediate intercellular communication within the oral microenvironment by influencing the behavior of immune cells, fibroblasts, and osteoclasts.^18^ Host-derived exosomes from macrophages, neutrophils, and gingival fibroblasts transport miRNAs that regulate macrophage polarization, shifting the balance between pro-inflammatory (M1) and anti-inflammatory (M2) phenotypes, which is critical for resolving or perpetuating inflammation.^81^ This polarization directly impacts the inflammatory milieu, with exosomal miRNAs such as miR-21 and miR-124 playing essential roles in promoting the anti-inflammatory M2 phenotype, thereby facilitating tissue repair.^82,83^ Furthermore, exosomal miRNAs modulate fibroblast functions, particularly by regulating matrix metalloproteinases (MMPs) and tissue inhibitors of metalloproteinases (TIMPs), thus influencing extracellular matrix degradation and remodeling.^84^ By targeting these diverse cell types, exosomal miRNAs regulate a complex network that drives the initiation, propagation, and resolution of periodontal inflammation and tissue destruction.

### 4.3 Bidirectional Interaction with the Oral Microbiome

Exosomal microRNAs are integral to the bidirectional communication between the host immune system and the oral microbiome.^26^ Within this microbial ecosystem, pathogenic species play a central role in driving dysbiosis and shaping host responses. In particular, red complex bacteria are strongly implicated in disease initiation and progression through their virulence factors that disrupt host–microbe homeostasis and promote chronic inflammation. Studies have shown that *P. gingivalis* and other periodontal pathogens can modulate the formation and miRNA content of host-derived exosomes, thereby affecting the host’s immune and inflammatory responses.^85,86^ Numerous *in vitro* studies have evaluated the interaction between LPS, particularly from *P. gingivalis*, and exosomal microRNAs, revealing their roles in regulating immune signaling, inflammatory cytokine production, and host–pathogen interactions.^70,72^ These microbial stimuli can influence the expression profiles of exosomal microRNAs implicated in TLR-mediated signaling, NF-κB pathway activation, and cytokine synthesis.^86^ Conversely, exosomal miRNAs secreted by host immune and epithelial cells may influence microbial physiology. Host-derived exosomal microRNAs have been reported to influence bacterial gene expression associated with adhesion, invasion, and virulence, thereby modulating the composition and pathogenic potential of the oral biofilm. This bidirectional communication demonstrates the role of exosomal miRNAs as mediators of cross-kingdom signaling, contributing to the delicate balance between microbial homeostasis and dysbiosis in periodontal tissues.^85^ Understanding this interaction opens new avenues for targeted microbiome-modulating therapies using engineered exosomes or miRNA mimics/inhibitors to restore periodontal health.

## 5 Conceptual Framework: From Molecular Footprints to Clinical Frontiers

To address the existing gap between molecular discoveries and clinical applications, we propose an integrative conceptual framework that illustrates the progression from exosomal miRNA biogenesis to their functional involvement in periodontal disease and their evolving clinical relevance. Although numerous studies have reported individual exosomal miRNAs involved in inflammatory signaling, immune modulation, and tissue remodeling, a unified model that links these molecular features with diagnostic and therapeutic strategies remains underdeveloped.

In this framework, dysregulated exosomal miRNAs such as miR-146a, miR-155, and miR-223 are highlighted as central regulators of host–microbe interactions and immune balance in the periodontal environment. These miRNAs, selectively sorted into exosomes, influence central signaling pathways including those related to TLR, NF-κB activation, and proinflammatory cytokines. Disruption of these regulatory circuits can lead to imbalanced immune responses, excessive inflammation, and consequent tissue degradation. Moreover, this framework emphasizes the translational implications of these findings. The detectability of exosomal miRNAs in noninvasive biofluids such as saliva and GCF underscores their promise as accessible biomarkers for early diagnosis and disease monitoring. Therapeutically, modulation of these miRNAs represents a novel strategy for targeted intervention, aligned with precision medicine approaches. The framework also integrates emerging systems-level methodologies, including advanced network-based analyses, bioinformatics tools, and multiomics platforms. These techniques offer a deeper understanding of miRNA–gene interaction networks and support predicting functional and clinical outcomes. A schematic representation of this integrative framework is illustrated in Figure 4.

**Figure 4:**
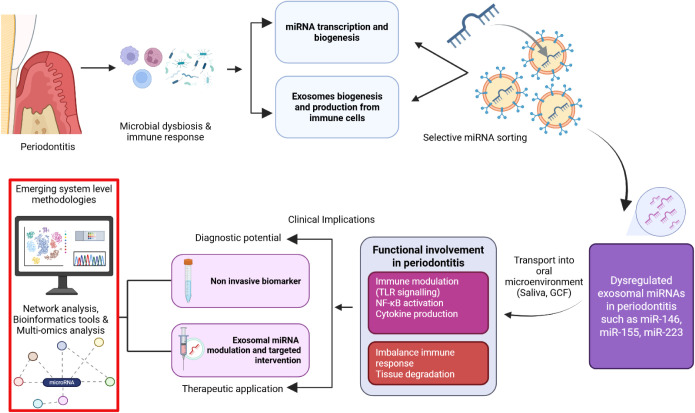
Integrative conceptual framework illustrating the role of exosomal miRNAs in periodontitis and their clinical potential. This schematic illustrates the progression from periodontal inflammation and microbial dysbiosis to the transcription of miRNAs and the biogenesis of exosomes by immune cells. These exosomes selectively incorporate specific miRNAs (miR-146a, miR-155, miR-223), which are transported into the oral microenvironment via saliva and gingival crevicular fluid (GCF). These dysregulated exosomal miRNAs modulate host–microbe interactions by influencing immune signaling pathways such as Toll-like receptor (TLR) and nuclear factor kappa-light-chain-enhancer of activated B cells (NF-κB), contributing to cytokine production, immune imbalance, and tissue degradation. Clinically, their presence in biofluids offers diagnostic value as noninvasive biomarkers, while their targeted modulation opens avenues for therapeutic intervention. The framework also incorporates systems-level methodologies, including bioinformatics, network analysis, and multiomics platforms to enhance translational potential and predict clinical outcomes. Created with BioRender.com.

## 6 Diagnostic Potential of Exosomal miRNAs

While exosomal miRNAs offer significant advantages over conventional diagnostic approaches, which often rely on clinical and radiographic assessments that identify periodontitis at advanced stages, their application is still in its early phases.^26^ Despite their promise, the reproducibility and validation of exosomal miRNA biomarkers across diverse populations remain challenging. However, their ability to detect periodontitis at a molecular level allows for early and precise intervention, potentially shifting the clinical diagnosis from a reactive to a preventive approach. By capturing disease progression before clinical symptoms appear, exosomal miRNAs enable timely therapeutic decisions and more effective treatment strategies.^87^

A key advantage of exosomal miRNAs is their noninvasive detectability in biofluids like saliva and GCF, making them valuable for diagnostic applications.^27,88,89^ Furthermore, they demonstrate disease-specific expression patterns, reducing the likelihood of false positives and negatives.^55^ This specificity is particularly valuable in distinguishing periodontitis from other oral inflammatory diseases, improving diagnostic accuracy and preventing unnecessary treatments. Our discussion focuses on selected and widely studied exosomal miRNAs, specifically miR-146a, miR-155, and miR-223, among the most frequently reported exosomal miRNAs implicated in periodontitis.^72,90,91^ A recent study by Memon et al. (2026), which evaluated miRNAs consistently associated with periodontitis across multiple studies, further confirmed that miR-146a, miR-155, and miR-223 are among the most reproducibly reported biomarkers. The meta-analysis demonstrated high diagnostic accuracy for miR-146a and miR-155, while miR-223 showed moderate performance, supporting their potential as noninvasive biomarkers for distinguishing periodontitis from healthy individuals.^92^

Emerging evidence indicates that exosomal miR-146a-5p may serve as a biomarker for periodontal disease severity, as its elevated expression in gingival tissues and saliva has been correlated with disease progression.^93^ However, its diagnostic consistency across different populations requires further validation. Jiang and colleagues (2020) demonstrated that miR-146a-5p levels were significantly higher in salivary small extracellular vesicles (sEVs) from periodontitis patients compared to controls, but not in whole saliva.^94^ In B cells, miR-146a-5p is upregulated by *P. gingivalis* LPS and targets the 3′ untranslated region (UTR) of IRAK1, inhibiting the production of inflammatory cytokines such as IL-1β, IL-6, and IL-10. Inhibition of miR-146a-5p increases cytokine production, while IRAK1 overexpression reverses these effects.^94^ This mechanistic role suggests that miR-146a-5p may represent a valuable diagnostic biomarker with a contributory role in periodontal pathogenesis.

Exosomal miR-155 has been widely investigated in periodontal disease, emphasizing its potential as a diagnostic biomarker and a key modulator of inflammatory responses.^95^ A recent study found a 5-fold increase in miR-155 expression in stage III/IV periodontitis, driven by NF-κB-mediated inflammatory signaling in gingival epithelial cells. This upregulation, potentially influenced by reduced IL-10 levels, exacerbates tissue damage and immune dysregulation in periodontal tissues.^96^ Additionally, elevated miR-155 levels were detected in the GCF of patients with chronic periodontitis, where it showed a positive correlation with superoxide dismutase activity, suggesting a link between oxidative stress and miR-155 expression. Given its ability to amplify inflammatory responses, miR-155 could be leveraged in diagnostic assays to stratify patients based on disease severity and risk of rapid progression. This suggests that miR-155 plays a significant role in the progression of periodontal disease and its association with systemic conditions such as Type 2 diabetes mellitus (T2DM).^97^ However, whether these associations are causal or merely correlative remains to be determined.

Exosomal miR-223 is crucial in regulating innate immunity, especially in inflammatory responses.^98^ While miR-223 plays a protective role in certain inflammatory diseases, in periodontitis, its overexpression promotes alveolar bone loss by targeting NF1-A, which triggers osteoblast apoptosis and facilitates osteoclast differentiation.^99^ This dual function, balancing immune regulation and bone resorption, highlights miR-223 as a key marker in differentiating between stable and progressing periodontitis cases, making it an essential component of molecular diagnostics. A recent study reported that elevated miR-223 levels are found in inflamed gingival tissues and their reduced expression during osteogenesis in PDL-derived cells. In periodontitis, miR-223 correlated with clinical parameters, and its overexpression inhibited osteogenesis by downregulating Fibroblast Growth Factor Receptor 2 (FGFR2) and Transforming Growth Factor Beta Receptor 2 (TGFβR2), key growth factor receptors.^71^ These findings indicate that miR-146a-5p, miR-155, and miR-223 are key modulators of inflammatory responses, positioning them as potential biomarkers of disease severity.

## 7 Translational and Therapeutic Potential

Given the multifactorial and complex nature of periodontitis pathogenesis, encompassing both inflammatory and tissue-destructive components, exosomal miRNA-based therapies offer innovative approaches to addressing the disease.^18,26^ The combination of regenerative and anti-inflammatory approaches represents a comprehensive strategy for the periodontitis therapy. Regenerative therapies, such as stem cell-based treatments and tissue-engineering approaches, focus on promoting tissue healing and regeneration.^100^ When integrated with anti-inflammatory therapies, such as those targeting pro-inflammatory cytokines or specific miRNAs involved in immune regulation, these strategies hold potential for addressing both the inflammatory and tissue-destructive components of periodontitis.^101^

The therapeutic potential of exosomes and their exosomal miRNA in periodontitis presents a novel avenue in regenerative medicine and disease modulation.^24^ Exosomal miRNAs modulate gene expression with high specificity, offering promising therapeutic potential for inflammatory conditions.^102^ One promising avenue is the use of exosome-based therapies to regulate inflammation, a key factor in periodontitis pathogenesis.^103^ Studies have demonstrated that exosomes derived from MSCs possess anti-inflammatory properties by modulating the expression of pro-inflammatory cytokines such as IL-1β, IL-6, and TNF-α through multiple mechanisms, including inhibition of NF-κB signaling, promotion of regulatory T-cell differentiation, and modulation of macrophage polarization.^104,105^ Targeting exosomal signaling pathways, such as NF-κB and TLR4, may also provide novel therapeutic strategies to mitigate periodontal inflammation and tissue destruction. These mechanisms are not merely passive anti-inflammatory effects but are crucial for reprogramming the inflammatory response in a manner that supports long-term immune homeostasis. Exosomal therapies contribute to the sustained resolution of inflammation by promoting the phenotypic shift of macrophages from a pro-inflammatory M1 state to a tissue-reparative M2 state.^106^ This reprogramming is important in chronic diseases such as periodontitis, where persistent inflammation results in continuous alveolar bone loss and periodontal ligament damage.

Another important translational aspect of exosomal miRNAs in periodontitis is their role in restoring immune balance.^107^ Notably, exosomal miR-155 has been implicated in modulating the Th17/Treg balance, a critical determinant of immune homeostasis in periodontitis. A study by Zheng et al. (2019) found a disrupted Th17/Treg equilibrium in periodontitis patients, with elevated Th17 and decreased Treg levels. Th17 cells promote inflammation, and Treg cells, which maintain immune tolerance, are key regulators of the immune response in periodontal disease. SIRT1, a protein involved in regulating inflammation, was found to be upregulated in exosomes from LPS-stimulated PDLSCs. These exosomes, with lower miR-155–5p and higher SIRT1 expression compared to normal PDLSC exosomes, alleviated inflammation through the Th17/Treg/miR-155–5p/SIRT1 network.^72,108^ This mechanism is particularly important because SIRT1-mediated deacetylation of NF-κB subunits directly suppresses pro-inflammatory cytokine production, reinforcing long-term immune regulation. These findings indicate that exosome-based therapies can achieve immune reprogramming at the molecular level, a crucial advantage over conventional anti-inflammatory drugs that primarily function by blocking inflammatory mediators without addressing the underlying immune dysregulation.^108^

Beyond immune modulation, exosomal miRNAs, including miR-223, miR-221–3p, and miR-222–3p, show potential in modulating osteogenesis and inhibiting osteogenic differentiation. Exosomal miR-223 suppresses osteogenesis in periodontal ligament cells by targeting growth factor receptors FGFR2 and TGFβR2, which are critical growth factor receptors involved in periodontal ligament cell differentiation, with elevated levels correlating with disease severity.^109^ This targeted inhibition prevents aberrant bone remodeling, which is a major challenge in periodontitis, where uncontrolled bone loss occurs alongside failed regeneration attempts. Similarly, exosomal miR-221–3p and miR-222–3p inhibit osteogenic differentiation of bone marrow mesenchymal stem cells under high-glucose conditions by targeting IGF-1 and modulating the IGF-1/ERK pathway, underscoring their role in impaired bone regeneration associated with diabetes.^109^ The further suppression of Wnt/β-catenin signaling by these miRNAs could be a significant mechanism in maintaining periodontal bone homeostasis, positioning them as promising candidates for therapeutic development.^110^

Recent bioengineering advancements have significantly enhanced the translational potential of exosome-based therapies for periodontitis. Techniques including the loading exosomes with specific therapeutic miRNAs, surface modification for targeted delivery, and large-scale production of functional exosomes have made their application in clinical settings more feasible.^111^ Hydrogel-based delivery systems have emerged as a promising solution, providing sustained and localized release of therapeutic exosomes within periodontal pockets.^109^ This localized retention is particularly critical in periodontitis treatment, as it ensures prolonged interaction of exosomes with host cells, enhancing their bioavailability and therapeutic effects. Hydrogels act as a protective matrix that stabilizes exosomes, preventing rapid degradation and ensuring their controlled release over time.^112^ Moreover, advanced hydrogel formulations allow for the incorporation of bioactive molecules that synergistically enhance the exosome function, further improving periodontal tissue regeneration and immune modulation. This dual approach aims to balance the need for tissue regeneration with the need to control chronic inflammatory conditions.^113^

The therapeutic application of exosomal miRNAs in periodontitis, however, remains experimental and requires careful consideration. Challenges related to the source, stability, dosage, and targeted delivery of exosomal miRNAs currently limit their clinical use. Addressing these barriers will require further preclinical studies and the development of advanced delivery systems to ensure their safety, reproducibility, and therapeutic efficacy.

## 8 Emerging Methodologies for miRNA Network Analysis

Advances in computational biology and systems-level approaches have significantly expanded our capacity to investigate the roles of exosomal microRNAs in the pathogenesis and progression of periodontal diseases. These methodologies provide enhanced precision in elucidating the complex regulatory networks that govern miRNA-mediated gene expression, thereby supporting both the mechanistic insight and translational relevance.

Bioinformatics tools and databases have been developed to facilitate the prediction, validation, and visualization of miRNA-target interactions. Platforms including miRNet enable the construction of disease- and tissue-specific miRNA–mRNA regulatory networks, supporting the analysis of context-dependent molecular interactions.^114^ Resources, including TargetScan, miRWalk and miRTarBase, employ predictive algorithms based on seed sequence complementarity, evolutionary conservation, and experimental validation to identify and confirm miRNA targets.^115,116^ Visualization tools, including Cytoscape, improve interpretability by integrating miRNA data with other omics layers, including transcriptomics and proteomics, into interactive network models.^117^

Beyond these individual tools, recent methodological developments have shifted toward system-level understanding through network-based and multiomics approaches. Competing endogenous RNA (ceRNA) networks, for example, model the interplay between miRNAs, long noncoding RNAs (lncRNAs), and circular RNAs (circRNAs), highlighting how these molecules compete for miRNA binding and regulate gene expression.^118^ Integrated omics strategies that combine transcriptomic, proteomic, and even microbiome data sets enable a multidimensional analysis of miRNA functions in disease-specific contexts.^119^ Additionally, predictive modeling using artificial intelligence (AI) and machine learning is increasingly applied to uncover novel regulatory circuits, prioritize biomarker candidates, and forecast disease progression or treatment response with improved precision.^120^ These approaches improve the accuracy of the disease classification, progression risk assessment, and treatment response prediction.

Together, these emerging methodologies represent a paradigm shift from isolated observations of individual miRNAs to a comprehensive and functionally integrated understanding of their roles in periodontitis.

## 9 Translational Challenges: Barriers and Solutions

Despite their potential, several challenges must be addressed before exosomes and exosomal miRNAs can be successfully integrated into routine clinical practice.^121,122^ One of the primary challenges lies in standardizing exosome isolation and characterization techniques, which significantly influence downstream applications and the reproducibility of results. Current methods for exosome isolation include ultracentrifugation, size-exclusion chromatography (SEC), precipitation-based kits, microfluidics, and immunoaffinity capture.^123^ Each method has distinct advantages and limitations, requiring careful consideration when selecting an approach for clinical or research purposes. Addressing these limitations presents a major opportunity for innovation as the development of automated, high-throughput, and cost-effective isolation techniques could accelerate clinical translation.

Ultracentrifugation remains the gold standard for exosome isolation due to its ability to produce relatively pure exosomal fractions. However, it is time-consuming, requires expensive equipment, and is often associated with low throughput and contamination by protein aggregates.^124^ In contrast, the SEC provides better separation of exosomes from protein contaminants and is gentler on exosomal structure.^123^ Nevertheless, they have limitations in scalability and may not be optimal for large clinical studies. Precipitation-based methods, often using commercially available kits, offer simplicity and convenience but lack the specificity required for isolating highly pure exosomes.^125^ Microfluidics-based techniques, while promising for their high sensitivity and ability to handle small sample volumes, are still under development for widespread clinical adoption and require further validation.^126^ Novel microfluidic devices, leveraging nanotechnology and AI, could improve the efficiency, yield, and specificity of exosome isolation while maintaining the scalability for clinical applications.

Characterization methods for exosomes include NTA, dynamic light scattering (DLS), electron microscopy (EM), and Western blotting to detect specific exosomal markers such as CD63, CD81, and CD9.^127^ These techniques, while robust, can vary in their sensitivities and specificities. NTA provides valuable information about exosome size and concentration but may be influenced by sample purity.^128^ EM offers high-resolution imaging but requires labor-intensive preparation and specialized equipment. Western blotting confirms the presence of exosomal markers but cannot assess purity or yield.^127^ To overcome these challenges, integrating machine-learning algorithms with high-throughput imaging and molecular profiling could improve the precision and efficiency of exosome characterization, enabling real-time analysis and quality control.

Biological variability further complicates the integration of exosomal miRNAs into the clinical practice. Factors such as age, gender, systemic conditions, and oral hygiene practices influence exosomal miRNA expression, potentially confounding their interpretation as biomarkers.^27,129^ Additionally, the heterogeneity of exosome populations and variability in miRNA profiles across individuals and disease stages create challenges in developing universal therapeutic strategies.^130^ However, this variability also presents an opportunity to develop personalized medicine approaches, where exosomal miRNA profiles could be tailored to individual patients, improving diagnostic precision and therapeutic efficacy. Advances in bioinformatics- and machine-learning-based biomarker discovery could aid in identifying patient-specific exosomal miRNA signatures, paving the way for precision medicine in periodontal therapy.

Translating exosomal miRNA research into clinical tools also requires thorough *in vivo* validation and longitudinal studies. To date, few studies have evaluated miRNA-based interventions in animal models or clinical trials, leaving a significant gap between laboratory discoveries and real-world applications. There is a need for well-controlled, multicenter clinical studies that rigorously assess the diagnostic accuracy, reproducibility, and therapeutic efficacy of exosomal miRNAs across diverse patient populations.

Another significant challenge involves the regulatory approval processes. As exosome- and miRNA-based treatments are relatively novel, the regulatory frameworks overseeing their clinical application continue to develop.^131^ Safety concerns, including the potential for off-target effects and long-term adverse outcomes, must be thoroughly evaluated. This is particularly crucial given the complexity of exosome production and the potential for variability in miRNA cargo.^132^ Establishing Good Manufacturing Practice (GMP)-compliant exosome production pipelines and standardized quality control measures will be essential to meet regulatory requirements and accelerate clinical adoption.

## 10 Future Directions

The identification of exosomes as multicomponent signaling entities facilitating intercellular communication, both within local environments and across distant sites, marks a rapidly advancing field of research.^133^ Salivary-derived exosomal miRNAs are progressing rapidly and hold significant potential for advancing novel strategies in the early detection, diagnosis, and personalized treatment of periodontitis.^134,135^ Distinct alterations in exosomal miRNA expression patterns have been reported between healthy individuals and those with periodontitis.^136^ These variations often correlate with disease severity, reinforcing their utility in monitoring disease progression and stratifying risk.^26^

Engineered exosomes hold significant potential as delivery vehicles for therapeutic agents, including anti-inflammatory drugs and functional anti-inflammatory miRNAs, offering a pathway for personalized anti-inflammatory therapies.^101,137^ Additionally, exosomes can be utilized to promote lysosomal degradation at sites of inflammation, enhancing therapeutic outcomes. Owing to their excellent biocompatibility, reduced toxicity, and low immunogenicity, exosomes offer a promising alternative for drug delivery.^101^ They exhibit remarkable stability in body fluids and can be loaded with specific molecules, enabling targeted delivery to specific cells for effective therapeutic intervention.^138^

Emerging strategies for exosomal miRNA-targeted therapies in clinical periodontal therapy focus on engineering exosomes to deliver miRNA mimics or inhibitors that regulate key inflammatory and regenerative pathways. Beyond direct therapeutic applications, exosome-based biosensors hold immense potential for precision diagnostics of periodontitis. Innovative hydrogel-based biosensors integrating CRISPR-Cas or aptamer-based technologies could facilitate real-time detection of specific exosomal miRNAs in saliva or gingival crevicular fluid, allowing early identification of disease progression and treatment response monitoring.^139,140^ These platforms could serve as point-of-care diagnostic tools, enabling clinicians to tailor interventions based on the patient-specific molecular signatures.

Additionally, novel hydrogel-based biosensors incorporating miRNA detection technologies are being explored to enhance point-of-care diagnostic capabilities. These hydrogels can be designed to capture exosomal miRNAs from saliva or GCF, providing rapid and minimally invasive diagnostics with high specificity. By integration of such biosensing platforms into periodontal care, clinicians could achieve real-time disease monitoring, significantly improving early intervention strategies.

Importantly, the integration of AI and systems biology is set to transform exosomal miRNA research. AI-driven modeling and machine learning classifiers can decode complex miRNA–gene regulatory networks, identify novel diagnostic signatures, and predict individual patient responses to treatment. However, successful clinical translation must account for substantial interindividual variability. Differences in genetic background, oral microbiome composition, systemic health status, and disease severity can influence exosomal miRNA expression patterns. This complexity highlights the limitations of universal biomarker panels and underscores the need for personalized miRNA signatures tailored to patient-specific profiles. Incorporating host–microbiome interactions and patient-specific variables into AI-based network analyses will improve the clinical relevance of biomarker discovery and interpretation.

Despite promising early findings, the clinical translation of exosomal miRNAs in periodontitis remains in its infancy. Large-scale, multicohort validation is significant to establish their diagnostic and therapeutic potential. Integrating exosome-based approaches with advanced biomaterials, scaffolds, or surgical interventions could redefine regenerative strategies; however, the mechanistic basis and optimal therapeutic combinations require further investigation. Moving forward, interdisciplinary collaboration among researchers, clinicians, and regulatory agencies will be essential to accelerating clinical adoption, ensuring that exosome-based innovations evolve from experimental concepts to transformative therapies in periodontology.

## 11 Conclusions

Exosomal miRNAs, particularly miR-146a, miR-155, and miR-223, have emerged as pivotal molecular mediators in the immunopathogenesis of periodontitis, bridging host responses, microbial interactions, and tissue remodeling. This review synthesizes current evidence into an integrative conceptual framework that transcends descriptive summaries, offering a mechanistic and translational roadmap from molecular footprints to clinical frontiers. Mapping the selective packaging, stability, and regulatory influence of exosomal miRNAs reveals their distinct potential for use as noninvasive diagnostic biomarkers and targeted therapeutics.

While salivary exosomal miRNAs demonstrate superior diagnostic specificity over traditional markers, their clinical translation remains constrained by technical variability, limited *in vivo* validation, and interindividual differences. Nevertheless, emerging tools such as network-based analytics, multiomics integration, and AI modeling are revolutionizing our capacity to decode miRNA-mediated networks in disease. Future directions should prioritize the development of engineered exosomes for targeted miRNA and anti-inflammatory delivery, alongside exosome-based biosensors for real-time, minimally invasive disease monitoring. Bridging the gap between molecular understanding and clinical application requires collaborative efforts in standardization, clinical trials, and regulatory alignment; nonetheless, the path forward is both essential and promising.

## Notes

No animal or human subjects by the authors were used in this study.

## Abbreviations

AI: Artificial Intelligence
Ago2: Argonaute 2
BMSCs: Bone Marrow Mesenchymal Stem Cells
BMBD: Bone Marrow–Derived Macrophages
CCL2: C–C Motif Chemokine Ligand 2
ceRNA: Competing Endogenous RNA
circRNA: Circular RNA
CVD: Cardiovascular Disease
DGCR8: DiGeorge Syndrome Critical Region 8
DLS: Dynamic Light Scattering
DM: Diabetes Mellitus
Dicer: Endoribonuclease Dicer
EM: Electron Microscopy
ERK: Extracellular Signal-Regulated Kinase
ESCRT: Endosomal Sorting Complex Required for Transport
Exos: Exosomes
FGFR2: Fibroblast Growth Factor Receptor 2
GCF: Gingival Crevicular Fluid
GECs: Gingival Epithelial Cells
GMP: Good Manufacturing Practice
GMSCs: Gingival Mesenchymal Stem Cells
HGFs: Human Gingival Fibroblasts
hDPCs: Human Dental Pulp Cells
hPDLSCs: Human Periodontal Ligament Stem Cells
IBD: Inflammatory Bowel Disease
IGF-1: Insulin-Like Growth Factor 1
IL-1β: Interleukin-1 Beta
IL-6: Interleukin-6
IL-8: Interleukin-8
IL-10: Interleukin-10
IL-17: Interleukin-17
ILVs: Intraluminal Vesicles
IRAK1: Interleukin-1 Receptor-Associated Kinase 1
lncRNA: Long Noncoding RNA
LPS: Lipopolysaccharide
MAPK: Mitogen-Activated Protein Kinase
miRNA: MicroRNA
MMPs: Matrix Metalloproteinases
MSCs: Mesenchymal Stem Cells
MVBs: Multivesicular Bodies
NF-κB: Nuclear Factor Kappa-Light-Chain-Enhancer of Activated B Cells
NF1-A: Nuclear Factor I-A
NG-PDLSCs-Exo: Normal-Glucose-Cultured Periodontal Ligament Stem Cell Exosomes
NOD: Nucleotide-Binding Oligomerisation Domain
NTA: Nanoparticle Tracking Analysis
nSMase2: Neutral Sphingomyelinase 2
PDL: Periodontal Ligament
PDLSCs: Periodontal Ligament Stem Cells
PRRs: Pattern Recognition Receptors
RA: Rheumatoid Arthritis
RANKL: Receptor Activator of Nuclear Factor Kappa-B Ligand
RBPs: RNA-Binding Proteins
RISC: RNA-Induced Silencing Complex
ROS: Reactive Oxygen Species
SIRT1: Sirtuin 1
SNPs: Single-Nucleotide Polymorphisms
SNARE: Soluble N-Ethylmaleimide-Sensitive Factor Attachment Protein Receptor
Spry1: Sprouty Homologue 1
TGFβR2: Transforming Growth Factor Beta Receptor 2
Th: T Helper Cells
TIMPs: Tissue Inhibitors of Metalloproteinases
TLR: Toll-Like Receptor
TNF-α: Tumor Necrosis Factor Alpha
Treg: Regulatory T Cells
UTR: Untranslated Region
VAMP7: Vesicle-Associated Membrane Protein 7
VDR: Vitamin D Receptor
Wnt5a: Wingless-Type MMTV Integration Site Family Member 5A
YBX1: Y-Box Binding Protein 1
